# Supplementary material for: Associated factors of health-related quality of life in Indonesian Women with Systemic Lupus Erythematosus: A cross-sectional within-cohort analysis
Source: PLoS One. 2026 Apr 24;21(4):e0341989. doi: 10.1371/journal.pone.0341989 (PMC13108768; doi:10.1371/journal.pone.0341989)
Supplement: Attachment — Submitted filename: Reviewer report to PLOS ONe-PONE-D-26-01165-20260303.docx [file pone.0341989.s002.docx]

**Manuscript Number:** PONE-D-26-01165

**Title:** Predictors of Health-Related Quality of Life in Indonesian Women with Systemic Lupus Erythematosus: A Cross-Sectional Within-Cohort Analysis

**General Comments**

This manuscript investigates the factors influencing health-related quality of life (HRQoL) in a specific cohort of Indonesian women with SLE. The study identifies sleep quality, anxiety, and age as significant independent predictors of QoL, explaining 36.1% of the variance. The finding that clinical disease activity (MEX-SLEDAI) and corticosteroid dose did not significantly correlate with QoL in this predominantly low-activity cohort is particularly noteworthy and highlights a potential "disconnect" between clinical markers and patient well-being. While the study is well-structured and uses validated instruments, several areas regarding statistical interpretation and local context require clarification to meet the standards of *PLOS ONE*.

### Specific Comments and Revision Requirements

#### 1. Methodology and Statistical Stability

- **Variable Definition:** The authors combine "Anxiety/Depression" into a single dichotomous variable. Given that the DASS-21 provides distinct subscales, please clarify the rationale for this grouping.
- **Subgroup Size:** Only 3 subjects (4%) were identified as having anxiety or depression. The authors must address the statistical limitations of drawing strong conclusions or high beta coefficients (-21.402) from such a small subgroup.
- **Multicollinearity:** Considering the known overlap between sleep disturbances and psychological distress, did the authors test for multicollinearity (e.g., Variance Inflation Factor) in their regression model?

#### 2. Clinical and Diagnostic Tools

- **MEX-SLEDAI Rationale:** The manuscript provides a strong justification for using the MEX-SLEDAI in resource-limited settings. To enhance the "Materials and Methods" section, explicitly state if specialized immunological assays (e.g., anti-dsDNA) were unavailable at the study site or if the tool was chosen for its practical utility in Southeast Asian clinical practice.
- **Pain Scale Thresholds:** The authors use a 40mm VAS cutoff to define "severe" pain. Please provide a specific citation supporting this threshold as a marker for functional impairment specifically within the SLE population.

#### 3. Results Interpretation

- **Variance Explanation:** The model explains 36.1% of HRQoL variance. The Discussion should more robustly address the remaining 63.9%, perhaps speculating on the roles of social support, cultural stigma, or permanent organ damage which were not measured in this study. It is recommended that the authors include theses study limitation in the discussion.
- **Education Levels:** In Table 2, the p-value for education is exactly 0.050. The authors should clarify if they consider this statistically significant or a trend, as the text describes it as having "influenced QoL".

#### 4. Discussion and Contextual Depth

- **Age and Quality of Life:** The finding that younger age predicts lower QoL is a highlight of the paper. The authors are encouraged to expand on the "burden of a life interrupted" by providing more specific local context regarding the social and professional expectations for young women in Indonesia.
- **Socioeconomic Factors:** 77.3% of the participants had incomes above the minimum wage. The authors should discuss how the recruitment from a private hospital clinic might skew the socioeconomic representation compared to the broader Indonesian SLE population.

#### 5. Actionable Recommendations

- **Intervention Specificity:** The conclusion advocates for "psychosocial and sleep interventions". To increase the paper's impact, the authors should briefly detail which components of Cognitive-Behavioral Therapy for Insomnia (CBT-I) and anxiety management are most feasible for implementation within the Indonesian healthcare framework.
