# Supplementary material for: Associated factors of health-related quality of life in Indonesian Women with Systemic Lupus Erythematosus: A cross-sectional within-cohort analysis
Source: PLoS One. 2026 Apr 24;21(4):e0341989. doi: 10.1371/journal.pone.0341989 (PMC13108768; doi:10.1371/journal.pone.0341989)
Supplement: Attachment — Submitted filename: Revision Letter and Responses.docx [file pone.0341989.s004.docx]

Dear Dr. **Gouda**,

Thank you for the opportunity to revise our manuscript. We are grateful to the reviewers for their constructive and insightful feedback, which has significantly strengthened the clarity and methodological transparency of our work.

We have addressed all the reviewers' concerns, particularly regarding the statistical treatment of the anxiety/depression variables, the sociocultural context of young women with SLE in Indonesia, and the feasibility of targeted sleep interventions. We believe these revisions have bolstered the clinical relevance of our findings for an international audience.

Specifically, we have:

- Clarified the statistical use of the DASS-21 and the reporting of its limitations.
- Added a "Sociocultural Context" section to the Discussion regarding Indonesian gender roles and HRQoL.
- Expanded on the "Intervention Specificity" by suggesting Brief Behavioral Treatment for Insomnia (BBTI).
- Provided formal collinearity diagnostics (VIF) to ensure model stability.

All changes in the manuscript have been tracked for your convenience. We hope the revised version is now suitable for publication in **[Journal Name]**.

Sincerely,

**Stevent Sumantri, MD, Ph.D.**

Corresponding Author

Division of Allergy and Clinical Immunology

Universitas Pelita Harapan / Siloam Hospitals Lippo Village

1. Variable Definition: The authors combine "Anxiety/Depression" into a single dichotomous variable. Given that the DASS-21 provides distinct subscales, please clarify the rationale for this grouping.

We thank the reviewer for this insightful comment. The decision to combine anxiety and depression into a single dichotomous variable was based on both clinical and statistical considerations.

First, in our cohort, the prevalence of clinically significant symptoms was low (n=3 for depression and anxiety). Notably, these cases overlapped entirely (comorbidity), making it statistically impossible to treat them as independent predictors in a multivariate model without causing significant multicollinearity issues.

Second, from an integrative clinical perspective, we aimed to identify a general 'psychosocial distress' signal. Given the high degree of internal consistency and correlation between DASS-21 subscales, this grouping allowed for a more robust and stable linear regression model, yielding a clearer β-coefficient for the impact of psychological health on HRQoL."

2. Only 3 subjects (4%) were identified as having anxiety or depression. The authors must address the statistical limitations of drawing strong conclusions or high beta coefficients (-21.402) from such a small subgroup.

We acknowledge the reviewer’s concern regarding the small subgroup (n=3) for the Anxiety/Depression variable and the resulting high beta coefficient. We agree that a beta of -21.402 is sensitive to the small number of participants in this category. However, the statistical significance (p=0.001) despite the small n indicates a very large effect size—specifically, a mean HRQoL difference of nearly 30 points (85.42 vs 56.66).

While we cannot claim this coefficient is a precise population constant, it serves as a robust indicator of the disproportionate impact psychological distress has on HRQoL in SLE. We have revised the manuscript to explicitly label this as a study limitation and have adjusted our language to be exploratory rather than definitive.

3. Considering the known overlap between sleep disturbances and psychological distress, did the authors test for multicollinearity (e.g., Variance Inflation Factor) in their regression model?

We thank the reviewer for highlighting the potential for multicollinearity between sleep quality and psychological distress. We agree that these domains often overlap in SLE populations.

To address this, we conducted a formal collinearity diagnostic during the model-building process. The **Variance Inflation Factor (VIF)** for all predictors in the final model ranged from [1.025] to [1.055], which is well below the conservative threshold of 2.5 (and the standard threshold of 10). Furthermore, the **Tolerance** values were all above 0.8. These results indicate that multicollinearity did not bias our regression estimates or the observed beta coefficients. We have added this detail to the 'Statistical Analysis' section of the revised manuscript.

4. MEX-SLEDAI Rationale: The manuscript provides a strong justification for using the MEX-SLEDAI in resource-limited settings. To enhance the "Materials and Methods" section, explicitly state if specialized immunological assays (e.g., anti-dsDNA) were unavailable at the study site or if the tool was chosen for its practical utility in Southeast Asian clinical practice.

We have revised the manuscript accordingly.

5. Pain Scale Thresholds: The authors use a 40mm VAS cutoff to define "severe" pain. Please provide a specific citation supporting this threshold as a marker for functional impairment specifically within the SLE population.

We have revised the manuscript accordingly.

6. The finding that younger age predicts lower QoL is a highlight of the paper. The authors are encouraged to expand on the "burden of a life interrupted" by providing more specific local context regarding the social and professional expectations for young women in Indonesia.

We appreciate the suggestion to expand on the impact of SLE on younger patients. We have added a section to the Discussion detailing the specific sociocultural pressures faced by young Indonesian women, particularly regarding marriage, fertility, and the dual expectations of professional and domestic roles, which contribute to the lower HRQoL observed in this demographic.

7. Socioeconomic Factors: 77.3% of the participants had incomes above the minimum wage. The authors should discuss how the recruitment from a private hospital clinic might skew the socioeconomic representation compared to the broader Indonesian SLE population.

The reviewer correctly notes the socioeconomic skew of our cohort. We have addressed this in the Discussion and Limitations sections by clarifying that our recruitment from a private hospital likely resulted in a higher-income representation. we have expanded on how this might 'buffer' some HRQoL impacts compared to the broader, more economically diverse Indonesian SLE population.

8. Do "Hours of sleep per night" ( in the Pittsburgh Sleep Quality Index) relate with QoL?

We thank the reviewer for this query regarding sleep duration. While the Global PSQI score was used in our primary model to capture the multidimensional nature of sleep disturbances (including quality, latency, and daytime dysfunction), we performed a post-hoc correlation analysis between 'Actual Hours of Sleep' (PSQI Component 3) and HRQoL.

We found that 'Hours of Sleep' alone did not significantly correlate with HRQoL (r = -0.221, p = 0.057). This suggests that in our cohort, sleep fragmentation and poor sleep quality—rather than sheer sleep deprivation—are the primary drivers of reduced quality of life. This aligns with existing literature suggesting that SLE-related fatigue is often non-restorative regardless of sleep duration."

9. Why male lupus is not investigated in this study ? ( Please explain it )

The decision to focus exclusively on female patients was made to ensure methodological and phenotypic homogeneity within a relatively small cohort (N=75)

10. This was a cross-sectional study so wont be able to establish the causal and effect relationship. Please revise the term “effects/predictors” to “association/associated factors”

We have revised the manuscript accordingly.

11. Please clarify whether the questionnaires were in English or Indonesian language. Please provide the validation study if the questionnaires were translated.

We appreciate the opportunity to clarify the linguistic validation of our instruments. All questionnaires used in this study (PSQI, DASS-21, and the HRQoL tool) were administered in the **Indonesian language**. We used previously validated Indonesian versions of these scales to ensure cultural and linguistic competence. Reference list updated accordingly.

12. What was the sampling method?

We have updated the sampling methods (consecutive) in the manuscript accordingly.

13. The objective also include the impact on daily function. What was the tool used to determine/ measure the daily function of the patients?

The reviewer is correct that daily function is a key objective. In this study, 'daily function' was assessed as a core component of the **Health-Related Quality of Life (HRQoL) instrument**. Specifically, we analyzed the [Physical Functioning/Activity] domains of the [Lupus QoL], which measures the extent to which health limits physical activities such as self-care, walking, and social roles.

14. The variables (sleep pattern, HRQoL, VAS, MEX-SLEDAI) were categorized. But in the statistical analysis, there was no chi square performed and/or multivariate logistic regression performed to determine the relationships between these variables.

We would like to clarify our statistical approach. While variables such as sleep quality and psychological distress were **categorized in the descriptive tables** (Table 1 and 2) to facilitate clinical interpretation (e.g., 'Poor' vs. 'Good' sleep), they were treated as **continuous variables** in the multivariate linear regression analysis. This approach was chosen to maximize statistical power and avoid the loss of information inherent in dichotomization. Therefore, a linear regression was appropriate to determine the predictors of the continuous HRQoL score, rather than a logistic regression.

15. Please clarify which one of the following was the dependant variable in this study. Sleep pattern or HRQoL?

We apologize for any ambiguity. In this study, **Health-Related Quality of Life (HRQoL)** was the **dependent variable**. Sleep patterns (PSQI), psychological distress (DASS-21), and clinical markers (MEX-SLEDAI) were treated as the independent variables (predictors). We have updated the manuscript accordingly.
